# Ergonomic Comparison of Four Dental Workplace Concepts Using Inertial Motion Capture for Dentists and Dental Assistants

**DOI:** 10.3390/ijerph181910453

**Published:** 2021-10-05

**Authors:** Daniela Ohlendorf, Laura Fraeulin, Jasmin Haenel, Werner Betz, Christina Erbe, Fabian Holzgreve, Eileen M. Wanke, Doerthe Brueggmann, Albert Nienhaus, Christian Maurer-Grubinger, David A. Groneberg

**Affiliations:** 1 Institute of Occupational Medicine, Social Medicine and Environmental Medicine, Goethe-University, 60596 Frankfurt am Main, Germany; j.lampe@med.uni-frankfurt.de (J.H.); holzgreve@med.uni-frankfurt.de (F.H.); wanke@med.uni-frankfurt.de (E.M.W.); brueggmann@med.uni-frankfurt.de (D.B.); Maurer-grubinger@med.uni-frankfurt.de (C.M.-G.); arbsozmed@uni-frankfurt.de (D.A.G.); 2 Department of Dental Radiology, Institute of Dentistry, Goethe-University, 60596 Frankfurt am Main, Germany; w.betz@em.uni-frankfurt.de; 3 Department of Orthodontics, University Medical Center of the Johannes Gutenberg-University Mainz, 55131 Mainz, Germany; erbe@uni-mainz.de; 4 Principles of Prevention and Rehabilitation Department (GPR), Institute for Statutory Accident Insurance and Prevention in the Health and Welfare Services (BGW), 20246 Hamburg, Germany; albert.nienhaus@bgw-online.de

**Keywords:** RULA, kinematic analysis, dental work concepts, dentist, dental assistant

## Abstract

When the inventory is arranged in a dental practice, a distinction can be made between four different dental workplace concepts (DWCs). Since the prevalence of musculoskeletal diseases in dental professionals is very high, preventive solution need to be investigated. As the conventionally used DWCs have, to date, never been studied in terms of their ergonomics, this study aims to investigate the ergonomic risk when working at the four different DWCs. In total, 75 dentists (37 m/38 f) and 75 dental assistants (16 m/59 f) volunteered to take part in this study. Standardized cooperative working procedures were carried out in a laboratory setting and kinematic data were recorded using an inertial motion capture system. The data were applied to an automated version of the Rapid Upper Limb Assessment (RULA). Comparisons between the DWCs and between the dentists and dental assistants were calculated. In all four DWCs, both dentists and dental assistants spent 95–97% of their working time in the worst possible RULA score. In the trunk, DWCs 1 and 2 were slightly favorable for both dentists and dental assistants, while for the neck, DWC 4 showed a lower risk score for dentists. The ergonomic risk was extremely high in all four DWCs, while only slight advantages for distinct body parts were found. The working posture seemed to be determined by the task itself rather than by the different inventory arrangements.

## 1. Background

**Figure 1:**
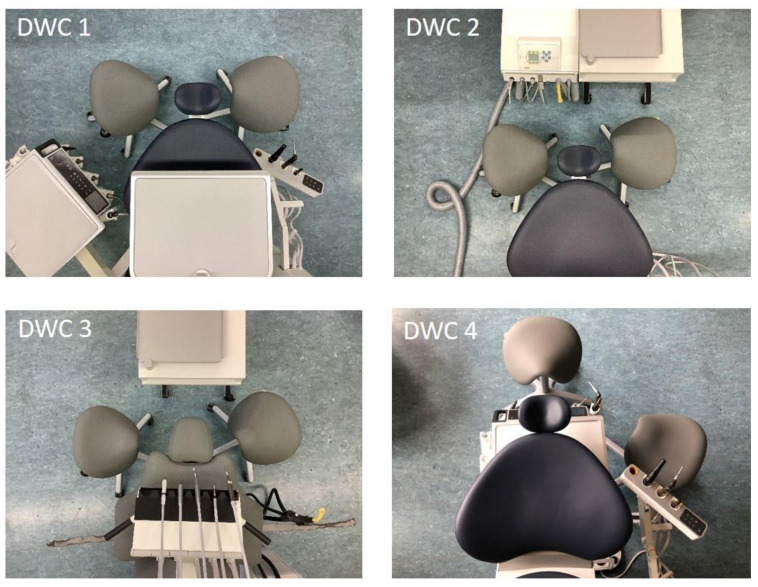
The different arrangements of the four dental workplace concepts (DWCs).

Awkward and static postures are known to be a major reason for work related musculoskeletal disorders (MSD) in the dental profession [1,2,3,4]. Dentists (Ds) and dental assistants (DAs) often sacrifice healthy postures in order to gain an optimal view into the patient’s mouth. When working in cooperative dentistry, the D as well as the DA need to have good vision of the working field, together with short reaching distances to the dental instruments and trays. These requirements are considered in the four commonly known dental workplace concepts (DWCs), which have been described by Kimmel [5,6,7], and are commonly sold by suppliers worldwide (Figure 1). DWCs differ in their positioning of the D and the DA in relation to the patient and the arrangement of the trays and instruments, while the patient lies in a supine position on the treatment chair in all four DWC. A detailed description of the DWCs can be found in the methodology under Dental Working Concepts (DWCs). These spatial prerequisites also determine the postures and movements of the dental professionals. While suppliers often argue about the ergonomic benefits of different DWCs, to date, no study has investigated the impact of these DWCs on the respective ergonomic risks.

An investigation of the realistic working conditions of Ds, as well as of DAs, also seems urgent, as a recent survey in Germany revealed that 92% of Ds [8] and 97.5% of Das suffered [1] from MSDs in at least one body region in the previous 12 months. MSDs are, besides being psychosocial factors, the major cause of ill health retirement in dentistry [9,10,11]. The body regions especially at risk in dental professionals are the upper extremities (shoulders and wrists), the neck, and the upper and lower back [1,2,3,8,12]. These body regions are well represented in the Rapid Upper Limb Assessment (RULA) [13], an internationally frequently used observation method for the classification of the ergonomic risks of working processes. RULA was originally designed as a paper−pen method, which was applied in order to quickly assess the potential health hazards in the workplace. In terms of dentists, this paper−pen method has already been used by Golcha et al. [14]. However, an evaluation of the complete working processes is difficult, and often only the most extreme postures are covered [15]. In order to obtain more detailed and objective information, Vignais et al. [16,17] suggested using the scoring system on inertial motion capture (IMC) data, which can be used in real work environments. IMC systems, such as MVN Link by Xsens (Enschede, The Netherlands), have been developed further recently and are now considered as accurate and reliable, especially for the investigation of slower movements [18,19], such as those found during dental treatment procedures [20].

As part of the SOPEZ project (Study for the Optimization Of Ergonomics In Dental Practice [4]), the aims of the current study were to analyze and to compare the ergonomic risks of the Ds and DAs when working together on the four known DWCs. Therefore, the working postures of generalists, orthodontists, oral and maxillofacial surgeons, endodontologists, and dental students were analyzed when working on the four dental quadrants. The nature of the present publication is explorative, but we sought to answer the following research questions: (a) is there a difference in the ergonomic risk of the four DWCs and (b) regarding each DWC, seperately, is there a difference in the ergonomic risk for Ds and DAs?

## 2. Materials and Methods

### 2.1. Subjects

In total, 75 dentists (37 male/38 female) and 75 dental assistants (16 male/59 female) volunteered to take part in this study. The Ds were 33.7 ± 8 years old (mean ± standard deviation), 175 ± 23 cm tall, and weighed 71.2 ± 11.9 kg. The DAs were 26.6 ± 5.4 years old, 154 ± 53 cm tall and weighed 67.1 ± 11.1 kg. Subjects were analyzed as pairs of one D and one DA. In order to gain a broad view on the dental working field, we analyzed 15 pairs (D + DA) each of generalists, orthodontists, oral and maxillofacial surgeons, endodontologists, and dental students. It should be noted here that students belonged to the group of general dentists. Subjects were only included if they were right-handed. In addition, they had to confirm that they had no current injuries to the musculoskeletal system (e.g., urgent herniated discs or slipped vertebra), severely restrictive malformations of the spine, rheumatic diseases, or stiffened spinal joints, and that any relevant surgery had taken place at least two years previously. This study was approved by the ethics research committee of the Goethe University (356/17) in Frankfurt am Main, Germany.

### 2.2. Dental Working Concepts (DWCs)

In dental practice, four DWCs are commonly known and used for the cooperative work of Ds and DAs (Figure 1), which were originally described by Kimmel [5,6,7]. In all four DWCs, both the D and the DA are seated close to the patient who is lying in a supine position. The D and DA are considered as a treatment team in all four DWCs, and teamwork is required for each of them. For the current investigation, the four DWCs were installed in a laboratory setting at the Institute for Occupational Medicine at Goethe University Frankfurt/Main (Germany). All DWCs were supplied with air pressure and water, while all necessary tools functioned as in normal dental practice.

*DWC 1* is based on the working concept developed by the German, Fritz Schön [21], in which the D sits at the 9 o’clock position to the patient, having a direct view into the patient’s mouth while reaching to the right for the instruments. The DA sits opposite the D in the 3 o’clock position, reaching to the left for instruments and to the right for a cupboard. The instruments are placed relatively low, while the tray is based above the chest of the patient.

*DWC 2* is predominantly based on Kimmel’s ideas [5,6,7]. In this DWC, the D also sits at the 9 o’clock position, but reaches to the left for the instruments; the DA sits opposite the D (as in DWC 1) and reaches to the right for instruments and hands the hose-free instruments to the D, which emphasizes the teamwork aspect of DWC 2. The instruments are also positioned relatively low in this DWC.

*DWC 3* is composed according to the suggestions of Kilpatrick (USA) [22], Guastamacchia (Italy) [23] and Skovsgaard (Denmark) [24]. Here, the instruments are placed higher than in DWC 1 and 2, hanging above the patient’s chest, which offers the shortest reaching distances of all DWCs for both the D and DA. The D, again, sits at the 9 o’clock position and the DA sits at the 2 o’clock position; both have to reach the instruments closely together, which makes the optimal positioning of the instruments crucial.

In *DWC* 4, which was developed by Beach (USA/Japan) [25], the hose-bound instruments are positioned in the treatment chair underneath the patient’s shoulders while the tray hangs above the patient’s chest. The D sits at the 12 o’clock position behind the patient’s head and has a partial direct view, but mostly indirect view, of the working field. The DA sits at the 2–3 o’clock position. Here, the work process requires a good communication and close teamwork at all times.

### 2.3. Measurement System

All kinematic data were obtained using the inertial motion capture system MVN LINK by Xsens, which has a sampling rate of 240 Hz. As there were scarcely any high speed movements, we chose a sampling rate of 24 Hz for the applied analysis, which has been described previously in more detail by Ohlendorf et al. [4].

### 2.4. Rapid Upper Limb Assessment (RULA)

**Figure 2:**
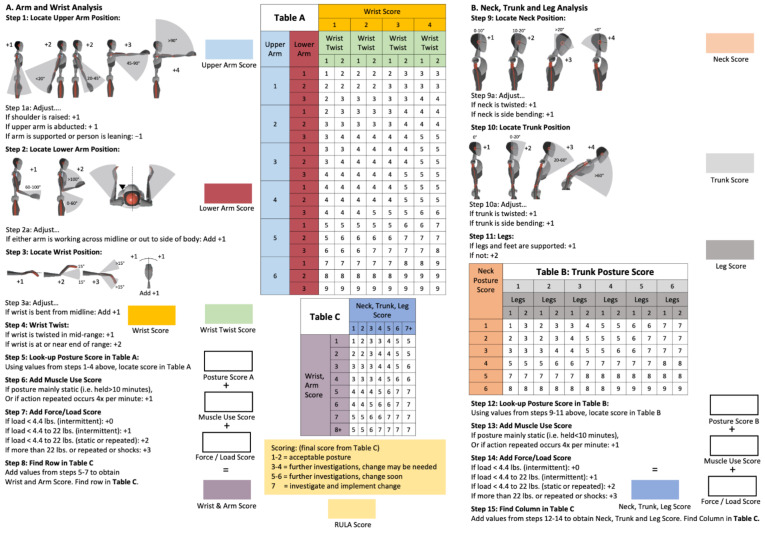
The Rapid Upper Limb Assessment (RULA) worksheet [27].

The RULA is an ergonomic risk assessment that was originally designed as a paper-pen−method in order to rapidly assess the ergonomic risk of potentially hazardous workflows [13]. RULA is composed of a scoring system in 15 steps, combining the joint angles of the upper limbs, neck, trunk, and legs with the weight-bearing, static, and dynamic working processes (Figure 2). In each step, the observer or company physician awards points according to the risk level, adding up to a final score, the value of which implies the urgency of changing the working process: score 1–2: acceptable risk; score 3–4: further investigation, change may be needed; score 5–6: further investigation, change soon; and score 7: investigate and implement change. The current analysis did not use the conventional paper−pen method, but an approach that determines ergonomic risk based on objectively collected kinematic data [26]. For this purpose, we assessed the final score in order to classify the total ergonomic risk of the dental working practice. As, in dental practice, the joint angles are of special interest and are frequently discussed as being a major health hazard, we chose to present them in order to analyze the postural risk between the body segments. For the right- and left-hand body sides, we chose step 1 (upper arm score), step 2 (lower arm score), and a combination of steps 3 and 4, which was suggested by Vignais et al. [16] (wrist score), as well as step 9 (neck position score) and step 10 (trunk position score). The maximum value to be scored for the neck, trunk, upper arm, and wrist was 6, respectively, while in the lower arm, a maximum score of 3 could be reached.

### 2.5. Measurement Protocol

After the subjects had put on the measurement suits and performed the calibration procedure, the treatment pairs (D and DA) performed a standardized procedure that reflected the most common dental treatments in each field of specialization; the dental students performed the same protocol as the generalists. Each protocol involved performing a dental activity in each of the four quadrants on a dummy head that was mounted on the treatment chair of each DWC. The exact treatment protocol for each field of specialization can be found in Ohlendorf et al. [4]. These standardized tasks were carried out for each of the four DWCs, taking 2–3 h in total to complete, depending on the working speed of the participants. Each team of Ds and DAs completed all their procedures on one single day. The working procedures of the Ds and DAs were recorded synchronously in the “No Level” scenario; this is a recording mode, available in the provider’s software, in which all joints and positions of the body segments are relative to the pelvis, not to the floor as is the usual norm. According to Xsens, this mode guarantees the best possible data accuracy and is the most suitable for ergonomic analyses [27].

### 2.6. Data Analysis

All recorded data were visually inspected for soundness by the authors, extracted from the software, and compiled to .mat files. All further analyses were conducted using a custom written script in MATLAB, version 2020a (The MathWorks, Inc., Natick, USA). This code consisted of the RULA worksheet, which was partly modified in order to apply it to the recorded objective kinematic data. Details on this script can be found in Maurer-Grubinger et al. [26]. For the current analysis, we chose different levels of complexity of the RULA analysis. First, the median RULA final score, being the most general and least complex evaluation, was determined. Subsequently, the RULA final score was regarded in more detail. As we took continuous recordings of the whole work processes, we were able to express how much time was spent relatively in each RULA final score value (possible: 1–7) as a relative average risk score over the time. This enabled a more distinct view on how risky the entire workflow actually is. Furthermore, we analyzed the kinematic RULA steps as well in median and in relative average risk score over the time.

### 2.7. Statistical Analysis

As the majority of the data were not normally distributed, we used the median values of the RULA scores. In order to consider the entire working process, we also calculated a relative average risk score over the time (rel. av. RST), meaning that we multiplied the relative time values with the RULA scores and added them (e.g., 0.3 × RULA 4 + 0.7 × RULA 5 = 4.7 relative average risk score over the time).

For Ds and for DAs, respectively, a comparison between the four DWCs was conducted. The relative average risk score over the time was applied to the Friedman Test, with a subsequent Conover-Iman comparison and a Bonferroni-Holm correction in order to compare the DWCs. Furthermore, in order to investigate potential differences between the Ds and DAs, we applied a Mann-Whitney U-test (and a Bonferroni-Holm correction) on the median scores and the relative average risk score over the time in each DWC, respectively. The significance level was set at α = 5%. In the kinematic step analyses, we also calculated how high the relative average risk score over the time was as a percent of the maximum RULA score possible (ergonomic risk potential (ERP)).

## 3. Results

### 3.1. Dental Assistants

**Figure 3:**
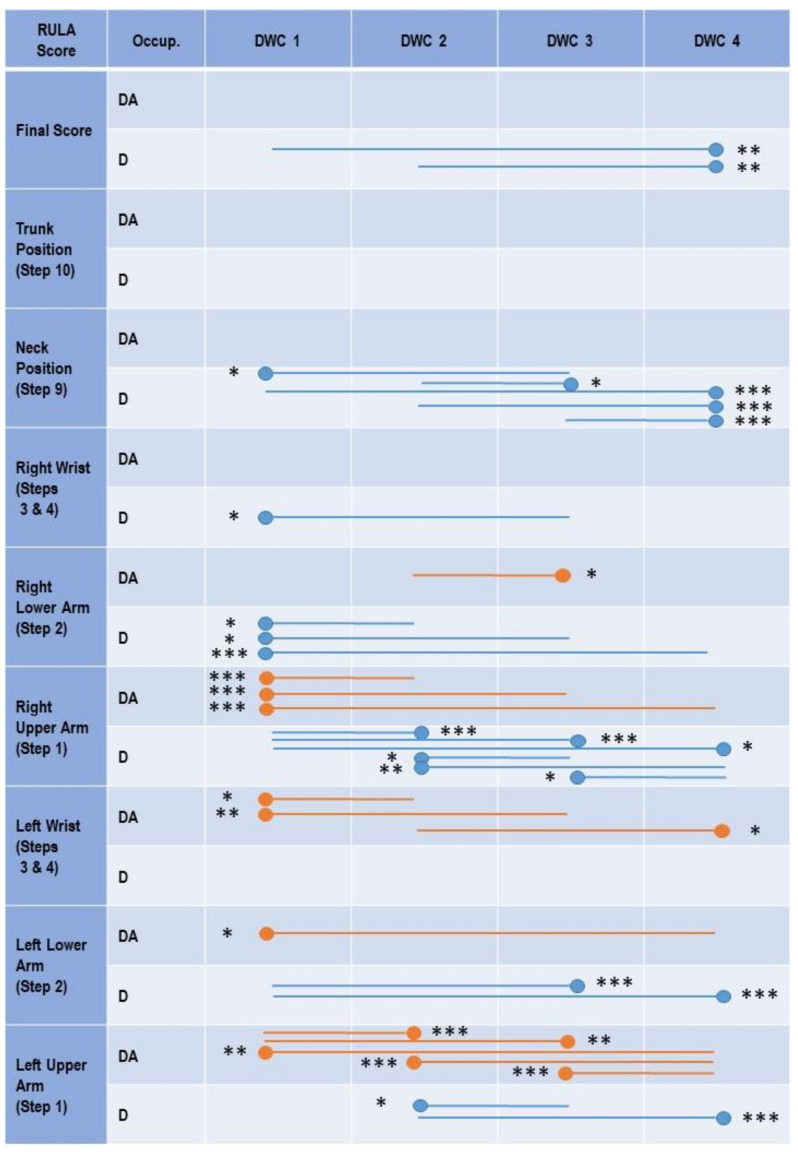
Significant differences of the relative average risk score over the time between the four DWCs for DAs (orange lines) and Ds (blue lines) in the outcomes: final overall, trunk score, neck score, wrist score left and right, lower arm score left and right, and upper arm score left and right. Significant differences are marked with lines indicating the relevant DWCs. Significance level: * ≤ 0.05, ** ≤ 0.01, *** ≤ 0.001. The circle marks the DWC with the significantly lower ergonomic risk score. Occup.— occupation; DWC—dental workplace concept; D—dentist; DA— dental assistant.

Here, no clear superiority of one DWC occurred in the final score (Figure 3), rather, the ergonomic risk was high in all four DWCs as the relative average risk score over the time reached 96.4–96.9% for the ERP (Table 1). Regarding the kinematics of the body segments, the ergonomic hazards were similar to those found in dentists—in both the lower arms and wrists (71.1–85.3%), the ERPs were highest, followed by the neck (58.7–59.9%) and the upper arms and the trunk (23.3–39.6%). Comparing the kinematics of the body segments among the DWCs, subtle differences were found. DWC 1 (26.4% ERP) proved to be favorable over the other DWCs (29.2–29.8% ERP) for the right upper arm. DWC 1 (71.1% ERP) also showed better results than DWCs 2 (73.4% ERP) and 3 (73.0% ERP) for the left wrist. In the left upper arm, however, DWCs 2 (23.3% ERP) and 3 (24.9% ERP) showed better results than DWCs 1 (26.6% ERP) and 4 (29.0% ERP).

### 3.2. Dentists

**Figure 4:**
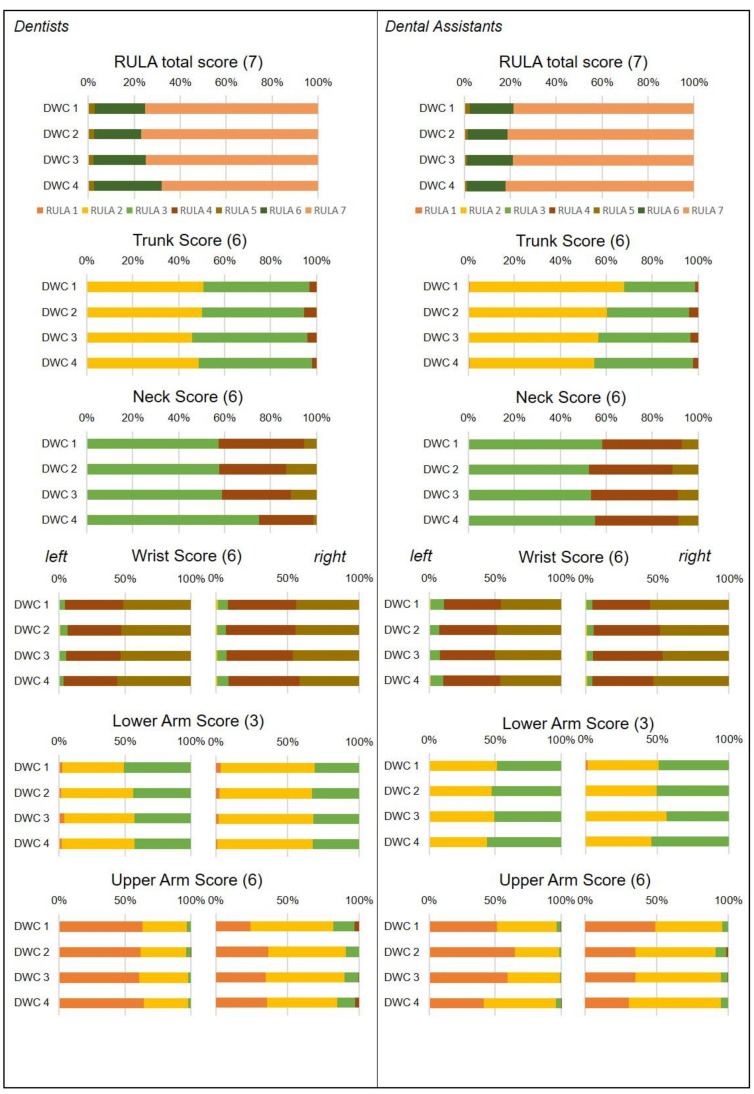
Relative average risk score over the time of the different RULA scores for all four DWCs. DWC—dental workplace concept. The maximum possible score of each body region is specified in brackets.

The relative time composition of the RULA final score showed that the risk is similarly high for all four DWCs (Figure 4). The ERP reached 94.9–96.29% (Table 1), indicating an urgency to change the working profile in all four DWCs. However, DWC 4 was slightly, but significantly, favorable compared with DWCs 1 and 2 (Figure 3). Analyzing the postural hazards of the dental work, the kinematic RULA steps revealed large differences between the body parts. Regarding the ERP (Table 1), the upper arms and the trunk showed the least ergonomic risk (23.5–42.9% ERP), followed by the neck (54.5–58.9% ERP) and both lower arms and wrists (71.6–82.1% ERP).

Although the data, again, showed similar values for all fours DWCs, statistically significant differences were found for some body segments. In the neck area, DWC 4 (54.5% ERP) was superior to the other three DWCs (58.2–58.9% ERP). For the right arm, more complex results were found—in the lower arm, DWC 1 (75.1% ERP) showed the least ergonomic risk (DWCs 2–3: 76.5–76.6 ERP), while showing the highest risk (32.8% ERP) in the upper arm (DWCs 2–3: 29.6–31.4% ERP). Here, DWC 2 (29.6% ERP) appeared to be slightly superior to the other DWCs. In the left lower arm, DWCs 3 (78.3% ERP) and 4 (77.2% ERP) were favorable over DWC 1 (82.1% ERP).

### 3.3. Differences in the Ergonomic Risk between Dentists and Dental Assistants by Dental Workplace Concept

The results of the Mann-Whitney U-tests are displayed in Table 1. In the following we describe only those findings that occurred in the relative average risk score over the time, not in the median values, as this allows for a more precise evaluation. In parentheses, we added ERP, followed by the *p*-value. In total, the results show that the ergonomic risks were mostly similar, but in some cases, slight, significant differences were revealed for the Ds and DAs.

### 3.4. DWC 1

In the final score, no differences occurred between the occupations, while the step analysis also did not clearly favor one group over another. In the trunk position, the DAs showed a significant lower risk (D: 41.8% ERP; DA: 37.8% ERP; *p* = 0.003); the DAs were also superior in the right upper arm (D: 32.8% ERP; DA: 26.4% ERP; *p* = 0.021), but, regarding the right lower arm, the Ds showed a smaller ERP (Ds: 75.1% ERP; DAs: 82.5% ERP; *p* < 0.001). This was also found for the right wrist (Ds: 71.6% ERP; DAs: 74.4% ERP; *p* = 0.02).

### 3.5. DWC 2

When working in this concept, the ergonomic risks were very similar between the Ds and DAs. Only regarding the right lower arm did the Ds show a slight advantage over the DAs (Ds: 76.6% ERP; DAs 82.7% ERP; *p* < 0.001), while in the trunk, the DAs had a slightly lower ERP (Ds: 42.7% ERP; DAs: 39.6% ERP; *p* = 0.04).

### 3.6. DWC 3

In DWC 3, the ERP in the right lower arm was higher in the Ds than in the DAs (Ds: 76.5% ERP; DAs 80.6% ERP; *p* < 0.001), and showed a similar tendency in the left lower arm (Ds: 78.3% ERP; DAs: 82.8% ERP; *p* = 0.014).

### 3.7. DWC 4

In this workplace arrangement, the Ds showed, clearly, a lower ERP than their assistants (Table 1). In the RULA final score, a highly significant difference, although only a small total difference, in the ERP was found (Ds: 94.9% ERP; DAs: 96.9% ERP; *p* < 0.001). In the following body parts, the Ds were at a slightly smaller risk: the neck (Ds: 54.5% ERP; DAs: 59.0% ERP; *p* < 0.001), the right lower arm (Ds: 76.6% ERP; DAs: 83.7% ERP; *p* < 0.001), left lower arm (Ds: 77.2% ERP; DAs: 85.3% ERP; *p* < 0.001), and the left upper arm (Ds: 23.6% ERP; DAs: 29.0% ERP; *p* < 0.001). A slightly significant superiority for the DAs was found only in the left wrist (Ds: 75.2% ERP; DAs: 71.8% ERP; *p* < 0.007) and the right upper arm (Ds: 31.4% ERP; DAs: 29.2% ERP; *p* < 0.003).

## 4. Discussion

The aim of the current study was to classify the ergonomic risk for dentists and dental assistants while working together on four different DWCs. Therefore, the working posture adopted during the performance of dental activities in all four quadrants was the focus of the analysis. In order to be able to analyze the representative dental activities, different fields of specializations were selected. This analysis was conducted due to the high prevalence of MSDs in Ds and DAs [1,3,4,28,29,30,31,32,33,34]. We aimed to gain insight into the extent to which the working posture, when using the four different inventory arrangements (DWCs) currently offered at practices worldwide, contributes to this.

In the final RULA score, the median ergonomic risk for both Ds and DAs in all four DWCs was 7, the highest possible score, which, thus, recommends an investigation and subsequent implemented change of the present working behavior. The significant differences for the Ds between DWC 4 and 1 or 2 were only due to a lower relative average risk score over the time in the double-digit decimal range of DWC 4. When looking at the ergonomic risk potential, it can be seen that the highest possible RULA score was reached between 95–97% of the working time for dentists, as well as for the dental assistants.

Although there are scarcely any relevant differences between the DWCs in the final RULA scores, there are clear differences among the individual body segments in the ERPs. The ERP values indicate a difference in the strain between the body segments: both lower arms and wrists (71.59–82.1% ERP), followed by the neck (54.4–58.4% ERP) and the upper arms and the trunk (23.5–42.9% ERP). These figures are virtually identical for the Ds and DAs, as can also be seen in Table 1 and Figure 4.

The lower score for the Ds’ necks may be due to the fact that in DWC 4, the dentist is seated behind the patient (at the 12 o’clock position). Thus, in DWC 4, the neck flexes without conducting an additional rotational or lateral bending movement, as is the case in the other DWCs. Due to the low lateral flexion and rotation movements, DWC 4 should be recommended for those Ds who have, for example, neck complaints or intervertebral disc disease, especially in view of the fact that this posture is maintained for the entire professional life. DAs have less opportunities to improve posture as the position of the assistant’s chair, relative to the patient’s chair, is identical in every DWC (3 o’clock position; Figure 1). In addition, it must be taken into account that the DA must always adopt an appropriate upper body posture in order to maintain eye contact with the D. However, as the DAs do not need access to a direct view of the patient’s mouth, as they are only assisting the D, then they experience less “contortions” than the Ds in performing their role.

The statistically significant, but still rather minor, relevant differences of the lower and upper arm positions (left and right body side) indicate that the different positioning of the tray and the two dental chairs beside the treatment chair are important. In this term, the wrists are less affected—only the right wrist for the Ds and the left wrist for the DAs (Table 1).

The statistical comparison between the two occupations per DWC, however, does not provide a clear conclusion as to whether the dentists or dental assistants work within a lower final RULA score or in the sub-segments. One major difference between the DWCs influencing the ergonomics are the different locations of the instruments to which the Ds have to reach when, i.e., grasping for drillers, which can be from the dentist’s point of view laterally, above, below, or from behind the patient. One further difference is the location of the D and the DA relative to the patient. In DWCs 1, 2, and 3, the D sits at the 9 o’clock position (DWC 3: 9–11.30 o’clock) and the DA at the 3o’clock position. Instead, in DWC 4, the D sits at the 12 o’clock position and reaches for the instruments directly in front of them from the treatment chair. Therefore, in DWC 4, the right upper and lower arm movements tend to be different compared to the other DWCs (Table 1), whereby the differences here are a maximum of 0.3 of the relative average risk score over the time between the data. Eventually, this leads to a better result in the final RULA score compared to the DA who sits in the 3 o’clock position.

However, in DWC 4, the grasping space for the D is small, i.e., the range of movement for the D’s forearm in which the D can grasp instruments without moving their upper body or upper arm. This is due to a better working position of the neck, right lower arm, and left upper and lower arms. Despite this, the working position for the right upper arm and left wrist is worse than for the DAs.

In conclusion, the grip paths are strongly influenced by the arrangement of the respective components in the DWCs. Regarding the strain on the upper body, especially for the D, this can play a role depending on whether the D has to turn distinctly in order to grasp the instruments or whether they can reach the instruments with little turning. The distances are at their minimum when the instruments are positioned above the patient’s upper body or in the headrest of the patient’s chair (DWC 4).

As the differences between the DWCs are sometimes only nuanced, although statistically significant, these small differences can have a large impact on the individual, especially if they already suffer from MSD. For complaints in the trunk area, therefore, working within DWCs 1 and 2 (for Ds and DAs) could potentially prevent an increase in these complaints, whereas the neck in DWC 4 contains a lower risk over the duration of work activity (especially for Ds) compared to the other DWCs. In DWC 4, the practitioner sits behind the patient, which seems to be the crucial point.

De Sio et al. [35] conclude in their review that the asymmetric working posture (extreme head and neck flexion, trunk inclination and rotation towards one side, lifting one or both shoulders, increased curvature of the thoracic vertebral column, and incorrect positioning of the lower limbs) during treatment, combined with, on the other hand, the assumption of static body postures over a longer period of time, are responsible for the etiology of MSDs in Ds and DAs. This working posture, especially the neck flexion, causes a 1.6-fold increase in cervical disc compression and a four-fold increase in anteroposterior shear at a posture of 45° compared to the neutral position [36]. Thus, these increases in cervical disc compression and shear forces during flexion, lateral flexion, and rotational movements must also be taken into account [37,38]. In the present analysis, the ERPs show that in the neck area, in all DWCs, between 55–59% of the working time was spent in the maximum possible RULA score.

The present findings are all the more alarming as previous surveys have proved that the lifetime, 12-month, and 7-day prevalence of musculoskeletal disorders (MSD) in dentistry [1,3,4,29,30,31,32,33] is higher than in the general population [39]. The high prevalence of MSDs in dentists and dental assistants suggests that this is associated with their working conditions (i.e., a very high ergonomic risk in the final RULA score; Table 1).

An ergonomic risk assessment of work activities, e.g., via RULA, is an important factor in the field of risk analysis of workplaces and also in connection with the prevention of MSDs, as its findings provide the theoretical basis for assessing working conditions [20]. In the present study, this is the working environment, i.e., the assessment of the dental setting is conducted through the kinematic body positions. However, a limiting factor that must be taken into account here is that only the wrist movement can be recorded with the Xsens and not the fine motor movement of the fingers; furthermore, these fine differences in the joint angles are difficult to see with the naked eye in contrast with the present, high-resolution kinematic analysis.

The comparison between the DWCs based on the RULA evaluation with kinematic data shows that, in the final RULA score, all four DWCs pose similar (high) ergonomic risks. However, it is noticeable that the ERP differs between the segments; in the upper and lower arm and trunk, for example, it is lower than in the neck or wrist. This is independent of whether the DWCs are worked at by dentists or dental assistants. It seems that the working posture is determined by the activity per se and is independent of the inventory arrangement. The different arrangement of essential controls alone cannot cause a positive or negative change. Thus, these relationship-related measures, i.e., a changed arrangement of the inventory, do not change the risk score. Following the RULA guidelines, restructuring measures should be taken to reduce the potentially highest ergonomic risk (final RULA score). In this study, four DWCs were selected that are globally known and available for purchase. For our laboratory investigation, we analyzed only these concepts, although we are aware that under realistic practical conditions, these “pure” arrangements are not always maintained and mixed forms are also used. Apart from this, the working posture could be determined more by the working habits and ingrained movement patterns than by the different inventory arrangements. However, this hypothesis has to be investigated in future analyses. Moreover, it should be further investigated whether the work experience shows an influence on the postural assessment or whether a “good” or “bad” working posture is independent of it.

Furthermore, it has to be considered that this is a laboratory study in which the arrangement of the inventory corresponds to the four respective DWCs. It should be investigated in further studies whether conducting a study in the familiar workplace or by using an individually-idealized arrangement would produce different results.

Whether the risk of musculoskeletal disorders can be reduced by behavioural measures, such as strength training or ergonomic posture training during dental work, should be explored in future analyses. If the ergonomic risk cannot be significantly reduced by restructuring the inventory arrangement, then muscle strengthening should be employed to maintain these ergonomically poor working positions over long periods by means of a well-developed “muscle corset”, and thus counteract musculoskeletal complaints. In this context, Yiu et al. [40] were able to show a score improvement of 1.88 in the RULA and a 17% increase in neck muscle strength in dental health students following 10 weeks of resistance training. However, the isometric strength of the shoulder muscles and the symptom severity of the neck/shoulder region showed no improvement within this pilot study. Although this behavioral intervention approach has barely been touched upon, it holds promising potential and needs to be explored further in future analyses.

Hence, forces, repetition, and task duration could be critical risk factors when assessing manual tasks as the activities performed in dental practice. Hence, further studies using other risk assessment methods would be desirable, which could support and extend our results. Besides strength training as one opportunity to reduce the ergonomic risks in dental workplaces, there are additional ideas for further experiments, even if they could not be conducted within this study. One could adjust minor changes to each DWC and analyze to what extent the joint angles, especially of the torso and limbs, might positively improve the ergonomics. In addition, the duration could be analyzed in more detail, considering how long in a real working day is worked in these high-risk scores, and then implementing a reduction of working hours or a change of schedule to minimize the adverse effects of such a risky working environment. The question also arises whether a third team member would be a support, as a supplementary worker “may” reduce the strain significantly and have a huge impact on productivity. In this respect, of course, the additional costs for a supplementary worker must also be taken into account. Furthermore, the use of microscopes or magnifying glasses could reduce the risk associated with the dental workplace.

The equipment of the workplace is an important aspect in dentistry, as well as the behavior or habits of the D or DA. Here, it is advisable for the individual to determine, through training, their most favorable way of working, i.e., that which appears to be the least stressful as possible. However, this presents a fundamental problem (in Germany); on the one hand, ergonomics at the workplace are only taught in a rudimentary way at dental school and, on the other hand, systematic cooperation between the dentist and the dental assistant is lacking.

## 5. Conclusions

The analysis of the ergonomic risk of the working postures of dentists and dental assistants, in the context of the four commonly used dental workplace concepts worldwide, shows that there is a median of 7 in the final RULA score for both Ds and DAs in all four DWCs, with between 95–97% of dental jobs being worked at the highest ergonomic risk score. For trunk complaints, DWCs 1 and 2 (for Ds and DAs) are favored, while for neck complaints, DWC 4 has a lower ergonomic risk score over the duration of the work activity, especially for Ds. The differences between the Ds and DAs per DWC are diverse and are due to the seat positions and are based on where the instruments are positioned in relation to the D/DA. Basically, the working posture seems to be determined more by the working habits and ingrained movement patterns than by the different inventory arrangements.

## Figures and Tables

**Table 1:** Median, relative average risk score over the time, and maximum score for all evaluated RULA steps, dental workplace concepts (DWCs), and both occupations. Comparison between the occupations: the occupation with the significantly smaller ergonomic risk is marked with asterisks and bold font (* *p* < 0.05; ** *p* < 0.01; *** *p* < 0.001). The alpha error was adjusted according to Bonferroni-Holm.

RULA Score	Occupation	DWC 1			DWC 2			DWC 3			DWC 4		
		Median (IQD)	Rel. av. RST	ERP (%)	Median (IQD)	Rel. av. RST	ERP	Median (IQD)	Rel. av. RST	ERP (%)	Median (IQD)	Rel. av. RST	ERP (%)
Final (Max. Score 7)	DA	7 (0.5)	6.75	96.43	7 (0)	6.77	96.71	7 (0.5)	6.75	96.43	7 (1)	6.78	96.86
	D	7 (0.5)	6.71	95.86	7 (0.5)	6.73	96.14	7 (0.5)	6.74	96.29	7 (0) **	6.64 ***	94.86
Trunk Position–Step 10	DA	2 (0.5)	2.27 *	37.83	2 (1)	2.38	39.64	2 (1) **	2.33	38.86	2 (1)	2.32	38.72
	D	2.5 (1)	2.51	41.84	3 (1) *	2.56 *	42.74	3 (1)	2.56	42.60	3 (1)	2.57	42.90
Neck Position–Step 9	DA	3 (1)	3.52	58.71	3.5 (1)	3.59	59.90	3.5 (1)	3.57	59.42	3.5 (1)	3.54	58.99
	D	3 (1)	3.49	58.22	3 (1)	3.53	58.87	3 (1)	3.51	58.53	3 (0.5) ***	3.27 ***	54.49
Right Wrist–Steps 3 & 4	DA	4.5 (1)	4.47	74.44	4 (1)	4.33	72.12	4 (1)	4.37	72.88	4.5 (1)	4.38	72.96
	D	4 (1) *	4.30 *	71.59	4 (1)	4.37	72.77	4 (1)	4.36	72.72	4 (1)	4.31	71.85
Right Lower Arm–Step 2	DA	2.5 (1)	2.48	82.53	2.5 (1)	2.48	82.73	2 (1)	2.42	80.62	2.5 (1)	2.51	83.74
	D	2 (1) ***	2.25 ***	75.14	2 (1) **	2.30 ***	76.62	2 (1)	2.30 **	76.51	2 (1) ***	2.30 ***	76.60
Right Upper Arm–Step 1	DA	1.5 (1) ***	1.58 ***	26.37	2 (1)	1.78	29.60	2 (1)	1.79	29.82	2 (0.5)	1.75 *	29.22
	D	2 (0)	1.97	32.75	2 (1)	1.78	29.60	2 (0.5)	1.83	30.46	2 (0.5)	1.88	31.38
Left Wrist–Steps 3 & 4	DA	4 (1)	4.26	71.05	4 (1)	4.40	73.40	4.5 (1)	4.38	73.01	4 (1)	4.31 *	71.77
	D	4.5 (1)	4.44	73.95	4.5 (1)	4.39	73.09	4.5 (1)	4.45	74.19	5 (1)	4.51	75.21
Left Lower Arm–Step 2	DA	2 (1)	2.44	81.27	2.5 (1)	2.50	83.17	2.5 (1)	2.49	82.84	3 (1)	2.56	85.30
	D	2.5 (1)	2.46	82.12	2 (1)	2.38	79.43	2 (1)	2.35	78.25	2 (1) **	2.32 ***	77.20
Left Upper Arm–Step 1	DA	1..5 (1)	1.60	26.58	1 (1)	1.40	23.29	1.5 (1)	1.49	24.86	2 (1)	1.74	28.99
	D	1 (1)	1.41	23.50	1 (1)	1.47	24.44	1 (1)	1.47	24.50	1 (1) *	1.42 ***	23.58

DWC—dental workplace concept; Rel. av. RST—relative average risk score over the time; ERP—ergonomic risk potential; D—dentist; DA—dental assistant; IQD—interquartile distance. Significance level: * ≤ 0.05, ** ≤ 0.01, *** ≤ 0.001. Significant values are marked in bold.

## Data Availability

The data presented in this study are available upon request from the corresponding author.
